# Evidence-based practice confidence and behavior throughout the curriculum of four physical therapy education programs: a longitudinal study

**DOI:** 10.1186/s12909-023-04821-0

**Published:** 2023-11-07

**Authors:** Wendy Romney, Nancy M. Salbach, Susan B. Perry, Judith E. Deutsch

**Affiliations:** 1 Department of Physical Therapy and Human Movement Science, Sacred Heart University, Fairfield, CT USA; 2 Department of Physical Therapy, Rehabilitation Sciences Institute, Temerity Faculty of Medicine, University of Toronto, Toronto, ON Canada; 3 The KITE-Research Institute, University Health Network, Toronto, ON Canada; 4 Department of Physical Therapy, Chatham University, Pittsburgh, PA USA; 5 RiVERS lab, Department of Rehabilitation and Movement Sciences, School of Health Professions, Rutgers, The State University of New Jersey, Newark, NJ USA

**Keywords:** Evidence-based practice, Physical therapy education, Confidence, Self-efficacy, Skill

## Abstract

**Background:**

Evidence-based practice (EBP) is a foundational process taught in health professional education, yet it is unclear when EBP confidence and skills are obtained. Increases in EBP confidence and behaviors from the start of physical therapy programs to post graduation have been reported in studies that evaluated a single program or used non-valid questionnaires. This study aimed to describe changes in EBP confidence and behavior using validated questionnaires of students from four physical therapy education programs throughout their curriculum and one year post graduation.

**Methods:**

One hundred and eighty-one students from a potential pool of 269 (67.3%) consented to participate. Students completed the Evidence-Based Practice Confidence (EPIC) Scale and the Evidence-Based Practice Implementation Scale (EBPIS) at 6 timepoints: start of the program, prior to first clinical experience, after first clinical experience, at the end of classroom instruction, graduation, and one year post. Medians (Mdn) and 25th and 75th percentiles (P_25_, P_75_) were calculated for 42 (23.2%) students with complete data across all timepoints. Change between timepoints was assessed using Friedman’s test and Wilcoxon signed rank test with a Bonferroni correction for post hoc analysis.

**Results:**

There were significant changes in EPIC scores (p < 0.001) from enrollment (Mdn 50.0, P_25_, P_75_ 35.5, 65.9) to prior to first clinical experience (Mdn 65.5, P_25_, P_75_ 57.3, 72.5) and after the first clinical experience (Mdn 67.3, P_25_, P_75,_ 58.9, 73.2) to the end of classroom instruction (Mdn 78.6, P_25_, P_75,_ 72.0, 84.1). Significant increases on the EBPIS (p < 0.01) were only seen from after the first year of training (Mdn 15, P_25_, P_75,_ 10.0, 22.5) to end of the first clinical experience (Mdn 21.5, P_25_, P_75_ 12.0, 32.0).

**Conclusions:**

EBP confidence increased significantly after classroom instruction but remained the same after clinical experiences and at one year post graduation. EBP behavior significantly increased only after the first clinical experience and remained the same through graduation. Confidence and behavior scores were higher than were previously reported in practicing professionals. Ongoing assessment of EBP confidence and behavior may help instructors build appropriate curricula to achieve their outlined EBP objectives.

**Supplementary Information:**

The online version contains supplementary material available at 10.1186/s12909-023-04821-0.

## Background

Evidence-Based Practice (EBP) is the integration of best available evidence, clinical expertise, and patient preferences [1]. EBP can improve quality of health care and patient outcomes and is a foundational skill taught in health care professional education [2, 3]. Despite the over 30 years of focus on teaching EBP in professional education, inconsistencies in the curricular content remain and lack of effective methods to change attitude, knowledge, confidence, skill, and behavior have been reported [4, 5]. Educators emphasize the first 3 steps of EBP (ask, search, appraise) and may not formally teach steps 4 and 5 (integrate and evaluate) [2, 5]. These omissions may lead to barriers such as lack of confidence and lack of skills, that are commonly reported to interfere with using evidence in practice [2, 6, 7].

To improve teaching and learning of EBP, core competencies [2] and curricular guidelines [3] have been created. Health care profession educators developed 68 competencies that align with the 5 steps of EBP and suggested levels of comprehension for each [2]. The Academy of Physical Therapy Research of the American Physical Therapy Association, also developed EBP Curricular Guidelines for physical therapy educators [3]. This consensus document was intended to standardize the skills taught in EBP education and has 33 terminal behavioral objectives arranged by the 5 steps of EBP. It provided suggestions for content evaluation, content mapping, student assessment, and faculty preparation. The developers further proposed that EBP skills be continually taught with increasing complexity throughout professional education, so they can more easily be integrated in practice.

The Sicily statement recommended valid and reliable standardized assessment tools to evaluate student EBP attitudes, knowledge, self-efficacy, skill, and behavior [8]. These recommendations complement the EBP Curricular Guidelines. [3] The need to use psychometrically sound assessment tools that accurately evaluate EBP education has been previously highlighted [1, 2, 4, 9]. The assessment tools recommended measure both EBP self-efficacy and behavior. The social cognitive theory suggests there is a link between self-efficacy or confidence and behavior [10]. Self-efficacy refers to people’s judgement (confidence) that they have the capacity and skills to perform certain activities [10]. For example, the social cognitive theory would suggest that if a student has confidence and self-efficacy to perform EBP, they may be more likely to engage in EBP.

Several studies in rehabilitation have measured changes in self-efficacy and behavior following evidence based educational interventions for both students and practicing rehabilitation professionals [11–15]. Findings on the relationship between self-efficacy and behavior have been mixed [11–15]. Tilson et al. [11, 14, 15] reported increases in practicing physical therapists’ self-efficacy and behavior following training in evidence-based practice that was sustained over 2 years. McEvoy et al. 2011 [12] found physical therapy graduates confidence remained the same one-year post graduation while EBP behaviors decreased. Petzold et al. 2012 [13] found a weak negative correlation between self-efficacy and evidence-based behaviors following training on unilateral spatial neglect of practicing occupational therapists.

To date, few studies have considered the social cognitive theory to report changes in physical therapy students EBP knowledge, confidence, and behavior across their professional education [12]. Other studies report on knowledge and behavior without measuring confidence [16, 17] Limitations of those studies included use of unvalidated questionnaires [16] and assessment of a single cohort of students or students from a single university [17]. Studies that have assessed EBP knowledge and behaviors immediately post-graduation have found behaviors remain the same or decrease after graduation [12, 16]. A cross-sectional study found that most graduates used clinical experience as their primary source of EBP decision making [18]. A longitudinal, mixed methods study on physical therapy and occupational therapy graduates found a decline in the use of EBP overtime and that EBP was influenced by personal and peer experiences, client needs and resources [19].

Questions remain about when EBP confidence is acquired, the frequency of EBP behaviors, and if they are retained after graduation. Further, comparisons across different curricula, public and private institutions, and countries are not reported in the literature. The purpose of this study was to describe changes in EBP confidence and behavior among students from the time of enrollment in four university-based physical therapy education programs up to one year post graduation.

## Methods

### Participants

The aim of this four-year longitudinal study was to describe changes in EBP confidence and behaviors among physical therapy students and graduates. Physical therapy students enrolled in entry-level physical therapy education programs at four universities in 2014 were invited to participate. At three universities, we described the protocol during recruitment presentations and interested students signed informed consent forms. At one university, students consented via completion of the first survey questionnaire after receiving an introductory email. The Institutional Review Boards at Chatham University, Rutgers University (ID# Pro20140000413) and Sacred Heart University (#140722A) and the Research Ethics Board at University of Toronto (#30,645) approved this study.

### Setting

The universities, their location, characteristics, and number of students were as follows:

- University A (American, Private, Doctorate, Problem Based, Small Cohort Size 42).
- University B (American, Public, Doctorate, Traditional, Medium Cohort Size 65).
- University C (American, Private, Doctorate, Problem Based, Medium Cohort Size 67).
- University D (Canadian, Public, Master’s, Traditional, Large Cohort Size 95).

Problem based curricula is a student-centered approach, where students are presented with a messy, real-world problem that doesn’t have a single correct answer [20]. At Universities A and C, the problems are presented to small student groups in the form of a patient case. Students must research, apply their knowledge and skill, and then collaborate to define the problem and determine a solution, such as, analysis of examination results or development of a plan of care. An instructor (tutor) facilitates the learning process, debriefs after each case, but does not “lecture.” [20] Traditional curricula is instructor-led, uses a lecture-based format, and may use patient cases, small group work and class discussion [20]. Additional details of each university curriculum can be found in Supplementary 1.

University A had five dedicated EBP courses, starting with portions of Principles of Practice I and II, leading to Research I-III. Weekly article presentations that included searching, appraising, and determining levels of evidence are required during small group discussion and in didactic courses that include problem-based learning. Small student groups complete a systematic review of the literature as a final project. This program encouraged students to present evidence-based in-services during full-time clinical experiences.

University B had four dedicated clinical inquiry courses combining evidence-based practice with clinical reasoning, as well as EBP integrated experiences in other courses. Two foundation courses teach students how to read Clinical Practice Guidelines, Systematic Reviews, Randomized Controlled and cohort studies, using a case-based approach. In the third course groups use a PICO (Patient, Intervention, Comparison, Outcome) format to systematically review and appraise the literature, with a goal of becoming effective and efficient with the process. A poster presentation with a submitted abstract is the final product of the course. The fourth course is on Outcome Assessment. Clinical experiences encourage students to present evidence-based in-services. Integration of evidence with clinical content occurs throughout the program.

University C had four dedicated evidence in practice courses. One foundational course taught students how to search and appraise the literature. Two courses used a case-based approach to teach students to ask, search, appraise and integrate the literature. The final course included a capstone experience where students designed a research protocol with literature review and plan for data analysis without project implementation. EBP is integrated in the problem-based learning course work through twice weekly article presentations based on patient cases. Clinical experiences required students to complete an evidence-based case presentation as well as encourage an evidence-based in-service. Integration of evidence with clinical content is throughout the program.

University D had three dedicated research courses as well as EBP content (e.g., overview of EBP steps, levels of evidence) and activities (e.g., acquiring, appraising, summarizing, and applying literature on clinical topics) integrated throughout the program. By the end of the program students complete a capstone research project with faculty facilitation, involving protocol development, Research Ethics Board application, data collection and analysis, manuscript drafting and poster presentation. Students complete an evidence-based case study report based on one of their clinical internships.

### Data collection

Students’ EBP confidence and behavior were measured using the Evidence Based Practice Confidence (EPIC) Scale [21, 22] and the Evidence Based Practice Implementation Scale (EBPIS), respectively [23]. These scales were recommended as valid and reliable EBP assessment tools by delegates at the 2009 International Conference of Evidence Based Health Care Teachers and Developers in the Sicily Statement [8]. Health care professionals and students use the 11-item EPIC scale to rate their perceived confidence in implementing steps of EBP on an ordinal response scale that ranges from 0% (no confidence) to 100% (completely confident). Item-level scores are averaged to determine a total score of 0-100% [21, 22]. Validity and reliability has been established in physical therapy [21, 22]. The EBPIS is an 18-item scale that measures the extent to which EBP is implemented. The EBPIS is scored on a 5-point frequency scale by indicating how often in the past 8 weeks the item was performed. The scale ranges from 0 meaning “0 times” to 4 meaning “greater than 8 times.” The score is totaled and can range from 0 to 72, 0 meaning no times over the past 8 weeks and 72 meaning greater than 8 times in all questions in the past 8 weeks. Validity and reliability have been established in nursing [23].

Demographic questions and the EPIC and EBPIS were uploaded into Survey Monkey® and sent to participants via email. The questionnaires were completed at the start of the physical therapy program (T0), just prior to the first full-time clinical experience (T1), after their first clinical experience (T2), at the end of the classroom instruction (T3), at graduation (T4) and one year after graduation (T5). The EBPIS was not completed at the start of the program (T0) as implementation of EBP was not anticipated prior to the start of their professional degree. The effect of classroom instruction was captured T1 and T3 and clinical experiences was captured at T2 and T4. Each timepoint differed across universities and is highlighted in Supplementary 1. The end of classroom instruction (T3) varied the most across universities. Universities A-C completed classroom instruction in the beginning or middle of the third year, while university D completed classroom instruction near the end of year 2.

### Data analysis

Participants who completed all questionnaires at all five times points were included in the analysis. A Mann Whitney U was completed to compare sex, age, prior research experience and scores at all timepoints for the EPIC and EPBIS to determine any differences between the included participants who completed questionnaires across all timepoints (“completers”) and those who did not (“non-completers”).

For the participants who completed all questionnaires, the total EPIC and EBPIS scores were summarized using medians and 25th and 75th percentiles within and across universities as data did not meet assumptions of normality. Friedman’s tests were used to determine significant changes across classroom instruction and clinical experiences as well as one year post graduation respectively within and across all universities for both the EPIC and EBPIS. Post hoc analyses were conducted using a Wilcoxon signed rank test with Bonferroni’s correction. Data were compared to the previous timepoint. The Bonferroni’s Correction for the EPIC scale was alpha < 0.0083 (p = 0.05/6) and for the EBPIS was alpha < 0.01 (p = 0.05/5) based on the number of times the questionnaires were completed. The percentage of participants achieving the Minimal Detectable Change (MDC) at the 95% confidence level (MDC_95_) of the EPIC scale of 6.1% was evaluated at each timepoint by each university and between all universities to determine if observed changes exceeded measurement error [22, 24]. MDC has not been established for the EBPIS. A Spearman rank order correlation was run to determine if there was a relationship between evidence-based confidence and behavior using the EPIC and EBPIS.

## Results

One hundred and eighty-one students from a potential pool of 269 (67.3%) consented to participate. A total of 42 (23.2%) students completed the EPIC and 44 (24.3%) completed the EBPIS at all timepoints. Regarding completion of questionnaires at all 5 timepoints, University A retained 6/28 (21%), University B 8/42 (19%), University C 15/54 (28%) and University D 13/57 (23%) individuals (“completers”). The total number of students with EPIC scores at each timepoint was T0 n = 139, T1 n = 129, T2 n = 111, T3 n = 107, T4 n = 85, T5 n = 60. Those with EBPIS scores at each timepoint was: T1 n = 126, T2 n = 107, T3 n = 105, T4 n = 84, T5 n = 59.

The median age of the participants at baseline was 22 years (P_25_, P_75_. 21, 24). The majority of students were female (n = 35, 80%), held a bachelor’s degrees (n = 40, 91%) and had previous research experience (n = 23, 52%) (Table 1).

**Table 1:** Participant Characteristics at Baseline by University

Characteristics	University A (n = 6)	University B (n = 9)	University C (n = 16)	University D (n = 13)	All (n = 44)
Age* Mdn (P_25_,P_75_)	23 (22, 23)	22^a^ (22, 25)	21^b^ (21, 22)	23 (22, 24)	22^c^ (21, 24)
Gender n (%) Male Female	1 (17%) 5 (83%)	3 (33%) 6 (67%)	2 (13%) 14 (87%)	4 (31%) 9 (69%)	9 (20%) 35 (80%)
Highest Education n (%) High School Bachelors Masters	0 (0%) 6 (100%) 0 (0%)	1 (11%) 8 (89%) 0 (0%)	2 (13%) 14 (87%) 0 (0%)	0 (0%) 12 (92%) 1 (8%)	3 (7%) 40 (91%) 1 (2%)
Participation in Research n (%) Yes Data analysis Literature Review Study Coordinator	3 (50%) 3 (50%) 1 (18%) 0 (0%)	4 (44%) 3 (38%) 0 (0%) 1 (13%)	6 (38%) 3 (20%) 3 (20%) 1 (7%)	8 (62%) 7 (54%) 7 (54%) 3 (23%)	23 (52%) 17 (74%) 11 (26%) 5 (12%)

Abbreviations: Mdn, Median: P_25,_ 25th percentile; P_75,_ 75th percentile, *Age data were missing for 2 participants noted in superscript a: n = 8, b: n = 15, c: n = 42

There were no differences between the participants who completed all five questionnaires (“completers”) and those who did not complete all five questionnaires (“non-completers”) for age (U = 1918.5, p = 0.226), previous research experience (U = 2008.0, p = 0.410) and across all timepoint for the EPIC and EBPIS (p > 0.05) (EPIC T0 U = 1772.5, p = 0.225, T1 U = 1791.5, p = 0.858, T2 U = 1351.1, p = 0.559, T3 U = 1142.5, p = 0.156, T4 = 781.0, p = 0.283, T5 U = 369.5, p = 0.891, EBPIS T1, U = 1612.5, p = 0.433, T2 U = 1326.0, p = 0.803, T3 U = 1098.0, p = 0.160, T4 U = 800.5, p = 0.367, T5 U = 374.0, p = 0.805). There was a significant difference in sex between completers and non-completers, (U = 1776.5, p = 0.022) as there was a lower percentage of females in the non-completing group (completers 81%, non-completers 51%).

Table 2 shows the differences across timepoints on the EPIC scores among pooled completers, by university, and the percentage of completers that achieved the MDC_95_ (6.1%) [22, 24].

**Table 2:** Scores on the Evidence-Based Practice Confidence Scale (EPIC) by Timepoint and University (n = 42)

Timepoint	EPIC score Mdn (P_25_, P_75_)				
	University A (n = 6)	University B (n = 8)	University C (n = 15)	University D (n = 13)	All (n = 42)
Start (T0)	48.6 (24.8,72.9)	44.6 (14.6, 58.4)	49.1 (37.3, 67.3)	53.6 (36.4, 66.4)	50.0 (35.5, 65.9)
Prior to first clinical experience (T1)	68.2 (54.8,72.9) ^b^	60.0 (44.3, 68.0) ^b^	65.5 (57.3, 71.8) ^a,b^	65.5 (58.7, 79.1) ^a b^	65.5 (57.3, 72.5) ^a,b^
After first clinical experience (T2)	65.9 (57.1, 89.6) ^c^	63.6 (59.1, 76.1) ^c^	67.3 (58.2, 71.8) ^d^	71.8 (54.6, 79.1) ^e^	67.3 (58.9, 73.2)^a,e^
End of Classroom Instruction (T3)	77.3 (74.1, 84.1)^c^	71.8 (62.1, 85.1) ^c^	80.0 (77.3, 84.0) ^a,b^	78.2 (69.6, 85.5) ^a,b^	78.6 (72.0, 84.1) ^a,c^
Graduation (T4)	87.3 (83.9, 93.0)^c^	78.2 (64.1,82.3) ^d^	82.7 (80.0, 89.1)^d^	80.9 (76.8, 84.6)^d^	81.8 (79.8, 88.2) ^a,d^
One year post Graduation (T5)	84.1 (75.7, 92.1) ^f^	77.3 (59.1, 88.4) ^d^	81.8 (71.8, 88.2) ^e^	75.5 (68.2, 81.8)^e^	78.2 (70.5, 85.0) ^e^

Abbreviations: Mdn, Median: P_25,_ 25th percentile; P_75,_ 75th percentile

^a^p<0.0083 from previous timepoint

^b^ >75% of participants had an increase from previous timepoint greater than Minimal Detectable Change (MDC) of 6.1%points

^c^ 50–75% of participants increased greater than MDC

^d^ 25–49% of participants increased greater than MDC

^e^ <25% of participants increased greater than MDC

^f^ >50% of participants had a decrease greater than the MDC of 6.1% points

**Fig. 1:**
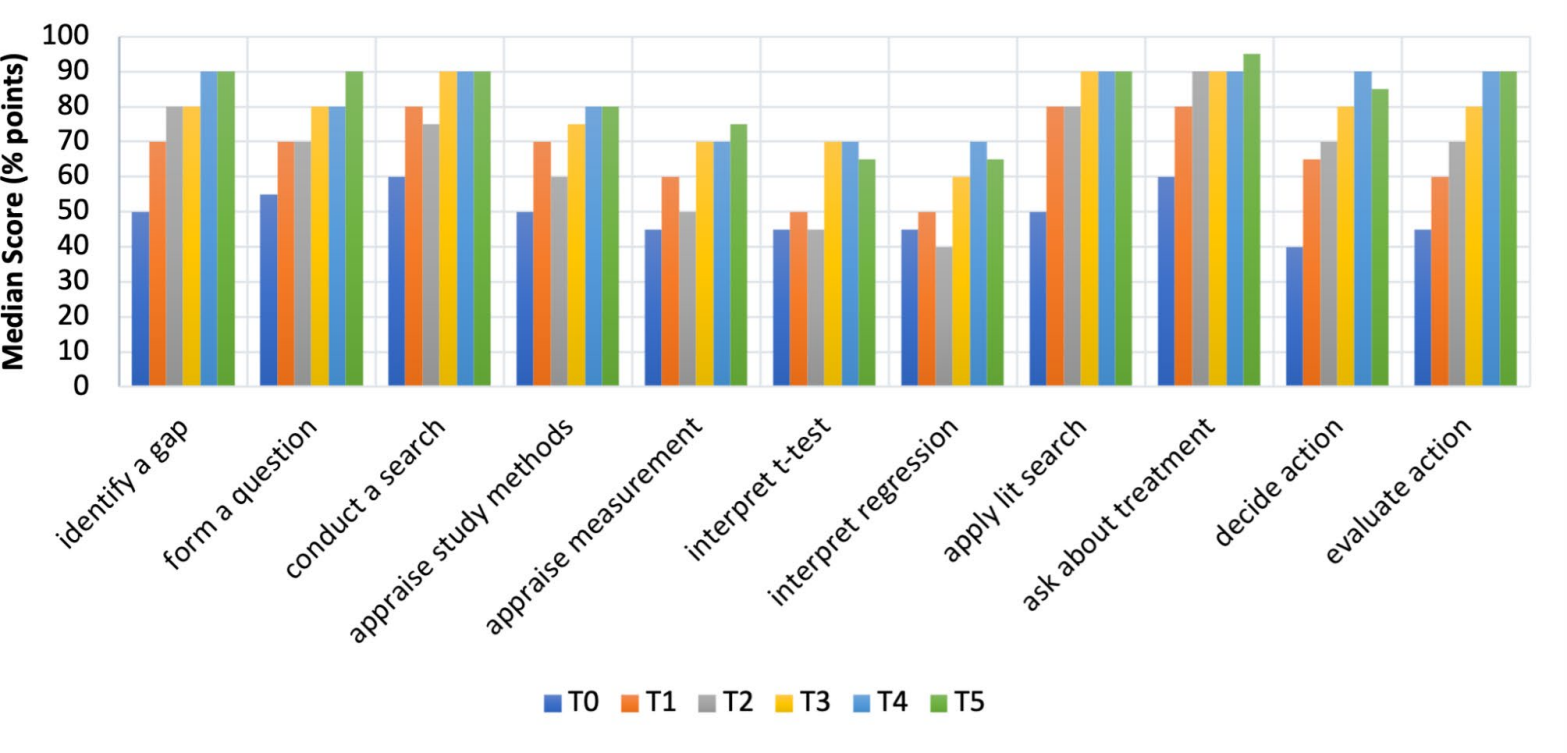
Median Scores on Evidence-Based Practice Confidence Scale (EPIC) Items by Timepoint. T0: Timepoint 0, start of program; T1: Timepoint 1, prior to first clinical experience; T2: Timepoint 2, after first clinical experience; T3: Timepoint 3, end of classroom instruction; T4: Timepoint 4, graduation; T5: Timepoint 5, one year after graduation

Figure 1 shows the individual items on the EPIC at start of the program (T0), graduation (T4) and one year post (T5). There was a statistically significant difference in EBPIS scores for pooled universities at T1-T2 only (after their first clinical experience) (chi square (4) = 22.611, p < 0.01, Z=-4.145, p < 0.001) (Table 3).

**Table 3:** Scores on the Evidence Based Practice Implementation Scale (EBPIS) by timepoint and university (n = 44)

Timepoint	EBPIS score Mdn (P_25_, P_75_)				
	University A (n = 5)	University B (n = 9)	University C (n = 17)	University D (n = 13)	All (n = 44)
Prior to first clinical experience (T1)	15 (8, 33)	12 (8, 25)	20 (14, 25)	12 (8, 18)	15 (10, 23)
After first clinical experience (T2)	23 (13, 28)	23 (13, 32)	30 (20, 40)	14 (11, 20)	21.5 (12, 32) ^a^
End of Didactic Training (T3)	21 (16, 27)	17 (16, 26)	23 (15, 32)	17 (8, 27)	21 (14, 28)
Graduation (T4)	12 (5, 24)	18 (12, 44)	36 (27, 43)	17 (10, 20)	21 (13, 38)
One year post graduation (T5)	20 (10, 24)	22 (14, 26)	22 (17, 35)	16 (9, 23)	20 (13, 25)

^a^ = < 0.001

Mdn: Median, P_25_: 25th percentile, P_75_: 75th percentile

When comparing within the universities on the EBPIS, none of the universities had significant differences when compared to their previous timepoint. Table 4 shows the median scores of individual items on the EBPIS prior to their first clinical experience, graduation and one year post graduation. There were weak positive correlations [25] between EPIC and EBPIS at T1 (rho = 0.245, p = 0.006) and T2 (rho=. 210, p = 0.03).

**Table 4:** Median Item and Total Score on the Evidence Based Practice Implementation Scale (EBPIS) prior to first clinical experience, Graduation and One Year Post Graduation (n = 44)

EBPIS Item	EBPIS Median Score		
	Prior to first clinical experience (T1)	Graduation (T4)	1 year post (T5)
1. Used evidence to inform my decision making	1	3	3
2. Critically appraised evidence from a research study	1	1	1
3. Generated a PICO question about a clinical case scenario	1	1	0
4. Informally discussed evidence from a research study with a classmate or instructor (colleague)	1	2	1
5. Collected data on a clinical case scenario	1	1	0
6. Shared evidence from a study or studies in the form of a report or presentation to greater than 2 classmates or instructors (colleagues)	1	1	0
7. Evaluated outcomes of practice decision	0.5	2	2
8. Shared a clinical practice guideline with a classmate/instructor/colleague	1	1	0
9. Shared evidence from a research study with a patient/family member	1	1	2
10. Shared evidence from a research study with a student or instructor from another discipline	0	1	1
11. Read and critically appraised a clinical research study	1	1	1
12. Accessed the Cochrane database of systematic reviews	1	1	0
13. Accessed the national guidelines clearinghouse	0	0	0
14. Used a clinical practice guideline or systematic review to make a clinical decision	1	1	1
15. Evaluated a care initiative by collecting patient outcome data	0	0	0
16. Shared the outcome data collected with classmates or instructors	0	0	0
17. Made a clinical decision based on patient outcome data	1	1	1
18. Promoted the use of EBP to my classmates or instructors (colleagues)	1	1	1
TOTAL score	14.5	20	18.5

0 = never, 1 = 1–2 times, 2 = 3–4 times, 3 = 5–8 times over the past 8 weeks

Abbreviations: EBPIS, Evidence Based Practice Implementation Scale

PICO: Patient/population, intervention, comparison, and outcomes

EBP: Evidence Based Practice

## Discussion

This longitudinal study on the EBP confidence and behaviors of physical therapy students and graduates across four physical therapy education programs found improvements in both EBP confidence and behavior. Specifically, EBP confidence increased significantly throughout the programs and remained the same at one year post graduation. EBP behavior increased the greatest after their first clinical experience and remained the same through graduation. This study assessed EBP confidence and behavior using recommended standardized tools throughout the curriculum. These results provide a basis for assessment throughout the curriculum as it can enhance planning to better prepare students to enact EBP.

Median EBP confidence generally increased from around 50–80% across the four universities. Both the pooled and individual programs had the highest percentage (> 50%) of participants exceeding the MDC_95_ after classroom instruction (T0 to T1 and T2 to T3). The 30% increase in confidence found in this study is similar to articles reporting on changes in occupational therapy and speech language pathology students following didactic and clinical education [26–29]. The occupational therapy and speech language pathology students started their programs with mean EPIC scores of 44% that increased to 78% at graduation [26–29]. Comparison with physical therapy education programs is not possible as the EPIC has not been previously reported in physical therapy students. Other authors have discussed increased confidence following didactic and clinical education using alternative scales throughout physical therapy programs and in didactic training in physical therapy practice [12, 16, 17].

The graduates in the present study generally rated their confidence on the EPIC higher (78–81%) than practicing rehabilitation professionals (53–70%) [14, 22, 30, 31]. This may be due to the lack of time that has elapsed since the participants completed their entry level EBP training. Similar to practicing rehabilitation professionals, participants in this study reported the highest confidence on the EPIC with EBP steps 1, 2, 4, and 5 and less confidence with appraisal (step 3) [22]. Salbach [22] reported scores higher than 70% on EPIC items 1–3 focused on asking and searching and EPIC items 8–11 on integration and evaluation. The participants in our study reported median scores 80% or greater on items 1–3 and 8–11. Median scores on items such as interpreting a t-test or a regression, in this study were 65%. Salbach [22] also reported low averages (35–39%) on these items.

The median EBP behaviors using the EBPIS had a significant increase between the end of the first year of classroom training (15/72) and the end of their first clinical experience (21.5/72). The improvement after clinical experience was unexpected as other authors have reported wide variations in EBP beliefs of clinical instructors, [32] multiple barriers’ to using EBP in the clinic [7] and the lack of communication between programs and clinical site regarding EBP and EBP Curricular Guidelines [5]. More research is needed to determine if clinical instructors are supporting students in their EBP behaviors.

Although there was an increase in EBPIS scores after the first clinical experience, these scores were low (15-21.5/72) and did not significantly change at subsequent timepoints through graduation and one year post. The low EBP behaviors reported in our study have been previously reported in nursing and occupational therapy students with total scores ranging from 14.5-25.34/72 [5, 33–35]. The EBPIS behaviors reported in our study were slightly higher than those reported among nurses and physical therapists with total scores ranging from 7.7–15/72 [36–38]. The most frequently reported behaviors (3–4 times over 8 weeks) of the students and graduates included using evidence to inform decision making, evaluating the outcome of practice, and sharing research with a patient or family. Participants, including graduates, reported never completing the following: *Accessing the National Guidelines Clearinghouse, evaluating a care initiative by collecting patient outcome data, and sharing outcome data collected with classmates/instructors/colleagues*. These behaviors align with step 5 of EBP and have been reportedly under-emphasized in education [2, 5].

In this study, each university differed in their methods of EBP content delivery and patterns of improvement, yet confidence and behaviors started and ended similarly across universities. Confidence improved the greatest across programs after classroom instruction (T0 to T1 and T2 to T3) and behavior improved after clinical experiences (T1 to T2 and T3 to T4). Confidence and behavior remained the same one-year post graduation. Chen [5] reported that the number of EBP credits, length of program, and number of program credits correlated with the number of EBP objectives and the expected mastery of the objectives in the EBP curricular guidelines. Class size inversely correlated with objectives and mastery. Interestingly, the four programs in this study varied in size, number of credits, and length, curricular structure and timing of didactic instruction and clinical placements yet EBP confidence and behaviors were similar. Future research should compare objectives and mastery with confidence and behavior.

Although the scales were scored differently, this study found relatively high confidence yet low frequency of EBP behaviors. There were weak positive correlations between EBP confidence and behavior at 2 timepoints, just prior to the first main clinical experience (T1) and after their first clinical experience (T2). Previous authors have reported the lack of relationship between evidence-based beliefs/confidence and evidence-based implementation using the Evidence Based Practice Belief Scale and the Evidenced Based Practice Implementation Scale for nursing students, [33, 35] occupational therapy students, [34] and practicing physical therapists [36]. Tilson et al. [15] provided a 6 month multi-component evidence based practice training to practicing physical therapists and found significant improvements pre- and post- with both the EPIC and the EBPIS scales, but did not report if there was a relationship between the two. The relationship between confidence and behavior using EPIC and EBPIS needs further investigation.

Many factors can influence EBP confidence and behavior that have not been captured in this study. These may include previous research experience, the emphasis (or lack thereof) on evidence in “non-EBP” (i.e. clinical management) courses, and the focus (or lack thereof) on EBP by clinical instructors during clinical experiences. These factors hint at the challenge of teaching the foundational EBP content and the potential need to emphasize integration of evidence across the curriculum as recommended by the curricular guidelines [3]. Future research should explore the most effective methods for delivering EBP content for professional health care students.

The acceptable level of EBP confidence and behavior among students, physical therapists, and rehabilitation professionals remains unclear. Physicians have reported they do not use all 5 steps of EBP in clinical practice [1]. Educators primarily teach steps 1–3 [2, 5]. Expecting graduate physical therapy students to be 100% confident in interpreting statistical analyses is likely not a reasonable goal for entry level practice education. Additionally, the low frequency of data collection reported on the EBPIS may not be a behavior we expect from students and rehabilitation professionals. The question remains about what evidence-based behaviors should be frequently completed. Such a determination may revise the weights of the scale items based on the ideal EBP practitioner behavior. Finally, relationships between EBP confidence and behavior, and patient outcomes, should be elucidated.

## Limitations

There were several limitations in this study. The response rate was low which reduces the generalizability of these findings. There was a high drop-out (attrition) rate (“non-completers”) that occurred across time, though this was similar to other previously reported longitudinal studies [16, 17, 19]. The highest level of drop-out occurred at one-year post graduation (T5). The between-school differences were reported descriptively and must be compared with caution due to the low number of completers. Participants at the university with the highest retention rate, and the highest EBPIS score, University C, were emailed the questionnaire at each timepoint from a familiar instructor (WR). This familiarity may have also biased these results.

## Conclusion

Evidence-based confidence and behavior improved and remained the same post-graduation following training in four physical therapy programs. A longitudinal comparison among several graduate physical therapy programs found greatest improvements in confidence after classroom instruction and greatest improvements in behavior after clinical experiences. Clinical experiences appear to improve the frequency of EBP behaviors, but this overall frequency remained low and remained low through the first year of practice. Assessment of EBP confidence and behavior may help instructors build appropriate curricula to achieve outlined EBP objectives. The acceptable level of EBP confidence and behavior across the five steps of EBP among rehabilitation professionals needs further investigation.

### Electronic supplementary material

Below is the link to the electronic supplementary material.

Supplementary Material 1

## Data Availability

The datasets supporting the conclusion of this article are available in the Harvard Dataverse repository at: https://dataverse.harvard.edu/dataset.xhtml?persistentId=doi:10.7910/DVN/WWAU7E [39].

## List of abbreviations

EBP: Evidence based practice
EPIC: Evidence-Based Practice Confidence
EBPIS: Evidence based practice implementation scale
Medians: Mdn, 25th and 75th Percentiles:P_25_, P_75_
PICO: Patient, Intervention, Comparison, Outcome
MDC: Minimal Detectable Change
