# Supplementary material for: Evidence-based practice confidence and behavior throughout the curriculum of four physical therapy education programs: a longitudinal study
Source: BMC Med Educ. 2023 Nov 7;23:839. doi: 10.1186/s12909-023-04821-0 (PMC10630997; doi:10.1186/s12909-023-04821-0)
Supplement: Supplementary file 1 — Supplementary Material 1 [file 12909_2023_4821_MOESM1_ESM.docx]

| University | Curricular Content Related to Evidence-Based Practice in 2015 | | | | | | | | | | |
| --- | --- | --- | --- | --- | --- | --- | --- | --- | --- | --- | --- |
|  | Year 1 | | | Year 2 | | | | | Year 3 | | |
| University A | **T0**  Principles in practice I (3 cr)  foundational lectures, PICO and critical appraisal assignments |  | Principles in practice II (3 cr): analyze diagnostic and intervention articles | **T1-T2**  Clinical: Options for in-service: content-based or focused on a particular line of evidence | Research 1 (3 cr): Foundational content to write a systematic review of the literature: 15 h of class time; 8 h of “journal club”; out of class meetings with instructor | | |  | Research 2 (2 cr): Continuation of Research 1: 6h class time; 2 “journal club”; 2 group discussion; 18 group work and out of class meetings with instructor; & meetings with course instructor | **T3**  Continuation of Research 2 (2 cr): out of class time to prepare SR presentation; 1h rehearsal; 4h class time for presentations | |
|  | *During 4 hours/week of problem-based learning, students must search for, find, critically appraise, and utilize appropriate levels of evidence during small-group clinical discussions* | | | *During 4-6 hours/week of problem-based learning, students must search for, find, critically appraise, and utilize appropriate levels of evidence during small-group clinical discussions* | | | | |  | | |
| University B | **T0**  Clinical Inquiry 1 (3 cr)  Case-based focus on intervention, CPG, SR, RCT | Clinical Inquiry 2 (2 cr)  Focus Prognosis and Diagnosis- apply RCTs | **T1-T2**  Clinical: Provided with EBP template | Clinical Inquiry 3 (2 cr): small group Practice Disseminate as poster | |  | Clinical Inquiry 4: (2cr) Outcomes chart audit, data analysis | | **T3**  Clinicals | | |
| University C | **T0** Introduction to psychometrics (5 hrs) | 622 (2 cr): foundational lectures  PICO and critical appraisal assignments | **T1-T2**  Clinical: EBP patient case presentation, Evidence based in-service | Grand Rounds (2 cr): EBP treatment case based | | Capstone 1 (2 cr): critical appraisal, develop research question | Clinical: EBP patient case presentation, Evidence based in-service | | Capstone 2 (2 cr): design a research protocol, lit review, planned data analysis | | **T3**  Clinical: EBP patient case presentation, Evidence based in-service |
|  | *During 6 hours/week of problem-based learning, students must search for, find, critically appraise, and utilize appropriate levels of evidence during small-group clinical discussions* | |  | *During 6 hours/week of problem-based learning, students must search for, find, critically appraise, and utilize appropriate levels of evidence during small-group clinical discussions* | | |  | | *Problem Based Learning: Tutorial Article Requirement* | |  |
| University D | **T0**  Unit 1/2: Foundational lectures (5 hrs): EBP steps, study design, PICO, levels of evidence, measurement, critical appraisal, systematic reviews, clinical practice guidelines  *Group assignment to search, review, and summarize literature for a cardiorespiratory condition; individual assignment to apply an article to plan assessment and treatment for a case scenario; individual critical appraisal assignment.* | Unit 3: *Group assignment to search and apply evidence to plan treatment for a given musculoskeletal population* | **T1-T2**  Unit 5: *Group assignment to search and apply evidence to plan treatment improve gait pattern of a neuro patient* | Unit 6 (3-wk unit dedicated to EBP): Lectures (24 hrs): research protocol development, ethics, study design, knowledge translation  *During ~54 hours of scheduled tutorial and independent work time, student groups develop and present a research proposal, draft a workplan, and submit an ethics application to undertake a capstone research project.* | Unit 8: 4 3-hr workshops (SPSS, Nvivo, qualitative data collection and analysis, quantitative analysis); 1 day/week (~15 days) for data collection.  Lecture (3 hrs) on program evaluation.  *3 Individual assignments to design a program evaluation of an existing clinical program; write a case report; and clinical reasoning presentation to address a patient care plan issue.* | | | **T3**  Unit 10/12 (6-wk unit dedicated to EBP): Lectures (3 hrs): manuscript writing, poster development, and publication/authorship.  *During ~200 hrs of scheduled independent work time, student groups analyze data, draft a manuscript and poster, and present poster at a research day.* | Not Applicable, this is a 2-year program | | |
| *Cr: Credits, hr: hours, PICO: patient, intervention, comparison, outcome, CE: clinical education*, CPG: clinical practice guideline, SR: systematic review, RCT: randomized controlled trial. T0: Timepoint 0, start of program, T1: prior to first full time clinical experience, T2: end of first clinical, T3: end of classroom instruction, T4: end of program. SPSS: NVivo | | | | | | | | | | | |

Supplementary 1: University Evidence Based Practice Course Sequence and Description
